# Detection of *BRAF*, *NRAS*, *KIT*, *GNAQ*, *GNA11* and *MAP2K1/2* mutations in Russian melanoma patients using LNA PCR clamp and biochip analysis

**DOI:** 10.18632/oncotarget.17014

**Published:** 2017-04-10

**Authors:** Marina Emelyanova, Lilit Ghukasyan, Ivan Abramov, Oxana Ryabaya, Evgenia Stepanova, Anna Kudryavtseva, Asiya Sadritdinova, Cholpon Dzhumakova, Tatiana Belysheva, Sergey Surzhikov, Lyudmila Lyubchenko, Alexander Zasedatelev, Tatiana Nasedkina

**Affiliations:** ^1^ Engelhardt Institute of Molecular Biology, Russian Academy of Sciences, Moscow, Russian Federation; ^2^ Blokhin Cancer Research Center, Ministry of Health of the Russian Federation, Moscow, Russian Federation; ^3^ P. Hertsen Moscow Oncology Research Institute, Moscow, Russian Federation

**Keywords:** biochip, somatic mutations, melanoma, diagnostic tool, targeted therapy

## Abstract

Target inhibitors are used for melanoma treatment, and their effectiveness depends on the tumor genotype. We developed a diagnostic biochip for the detection of 39 clinically relevant somatic mutations in the *BRAF*, *NRAS*, *KIT*, *GNAQ*, *GNA11*, *MAP2K1* and *MAP2K2* genes.

We used multiplex locked nucleic acid (LNA) PCR clamp for the preferable amplification of mutated over wild type DNA. The amplified fragments were labeled via the incorporation of fluorescently labeled dUTP during PCR and were hybridized with specific oligonucleotides immobilized on a biochip. This approach could detect 0.5% of mutated DNA in the sample analyzed. The method was validated on 253 clinical samples and six melanoma cell lines.

Among 253 melanomas, 129 (51.0%) *BRAF*, 45 (17.8%) *NRAS*, 6 (2.4%) *KIT*, 4 (1.6%) *GNAQ*, 2 (0.8%) *GNA11*, 2 (0.8%) *MAP2K1* and no *MAP2K2* gene mutations were detected by the biochip assay. The results were compared with Sanger sequencing, next generation sequencing and ARMS/Scorpion real-time PCR. The specimens with discordant results were subjected to LNA PCR clamp followed by sequencing. The results of this analysis were predominantly identical to the results obtained by the biochip assay. Infrequently, we identified rare somatic mutations.

In the present study we demonstrate that the biochip-based assay can effectively detect somatic mutations in approximately 70% of melanoma patients, who may require specific targeted therapy.

## INTRODUCTION

Melanoma is the most aggressive form of skin cancer, and the incidence of melanoma continues to rise worldwide [1, 2]. Although surgical treatment of early melanoma leads to 90% cure rates, unresectable advanced melanoma is notorious for its intrinsic resistance to chemotherapy, aggressive clinical behavior and tendency to rapidly metastasize. Five-year survival rates for patients with distant metastatic disease remain below 20% [2]. Therefore, despite the variety of approaches used to treat melanoma, novel therapies and treatment strategies are needed. In the past decade, targeted inhibitors have been widely used for the treatment of melanoma. The effectiveness of these treatments depends on the presence of driver mutations in the tumor.

Melanoma patients frequently harbor somatic mutations in a number genes, including the *BRAF* gene that encodes the serine-threonine kinase [3]; the *KIT* gene that encodes the receptor tyrosine kinase [4]; the *NRAS* [5], *GNA11* [6, 7] and *GNAQ* [7, 8] genes that encode the GTP-binding proteins; and the *MAP2K1* [9] and *MAP2K2* [10, 11] genes that encode the dual specificity mitogen-activated protein kinase kinases. The activating mutations in these genes lead to the constitutive activation of the RAS/RAF/MEK/ERK (MAPK) and the PI3K/PTEN/AKT (AKT) signaling pathways [7, 12–22]. Overall, mutations in these genes can be detected in approximately 70% of melanomas, depending on the site of the primary lesion [9, 23].

Tumor mutation status has been associated with the sensitivity of melanomas to specific targeted therapies. *BRAF* V600 mutations are linked with an increased sensitivity to *BRAF* (vemurafenib and dabrafenib) and MEK (trametinib and cobimetinib) inhibitors [24–32]. The type of *BRAF* mutation influences on sensitivity to targeted therapy. Thus, inhibition of BRAF with vemurafenib improves survival in patients with V600E and V600K mutations, but the response rate in patients with V600K was less than that in patients with *BRAF* V600E tumors [30]. The patients with rarer non-V600E *BRAF* mutations are showed objective responses to vemurafenib too; however, additional follow-up is required [33].

Tumors that harbor *KIT* mutations (W557R, V559D, K642E, L576P, and V559A) display a sensitivity to the KIT inhibitor, imatinib [4, 34–38]. The *KIT* V559A and L576P mutations are sensitive to the multi-tyrosine kinase inhibitor, sunitinib [39, 40]. Tumors that harbor the *KIT* L576P mutation show a response to tyrosine kinase inhibitors, such as dasatinib [41] and nilotinib [34]. Melanoma patients with *KIT* D816H mutation, however, are not sensitive to imatinib [35]. Furthermore, KIT kinase mutations D816H, D816V and D816Y show drug resistance to imatinib and sunitinib in gastrointestinal stromal tumor patients [35, 42–45]. Preclinical data suggest that MEK inhibition may be effective for uveal melanomas carrying *GNAQ* or *GNA11* mutations [23]. *MAP2K1/2* mutations confer resistance to MEK and BRAF inhibition [10, 11, 46, 47]. In most cases, mutations in the *BRAF, NRAS, KIT, GNAQ* and *GNA11* genes are mutually exclusive [8, 23, 35, 48] In contrast, *MAP2K1* and *MAP2K2* mutations often occur together with *BRAF* mutations [9, 46, 49].

Several techniques are currently used to detect somatic mutations in cancer cells. Among the most common techniques are Sanger sequencing, pyrosequencing, allele-specific real-time PCR and next generation sequencing (NGS). These methods have some advantages and disadvantages. The Sanger sequencing and pyrosequencing are inexpensive methods but have low analytical sensitivity and require setting the parallel reactions for each of the analyzed loci. The allele-specific real-time PCR is highly sensitive for the detection of any known mutation, but it is expensive and has the limitation of multiplex analyses. NGS has a high analytical sensitivity and allows the simultaneous detection of a large number of somatic mutations but is very expensive and time-consuming. Some of the approaches are fully automated [50–52], which makes them more attractive to clinicians but significantly increases the cost of analysis.

DNA microarray technology is successfully applied for the multiple testing of genetic markers in tumor cells. The high-density microarrays, for example, the OncoScan assay (Affymetrix, a Thermo Fisher Scientific company, Waltham, USA), allow simultaneous analysis of numerous genes, including structural, copy number and single nucleotide variations [53, 54]. The technology is very powerful and reliable but the high cost limits routine clinical use. Besides, the analysis is multistage and requires sophisticated bioinformatics. Low-density microarrays [55–60] allow to detect much less targets, however, they offer an inexpensive, fast and easy way to analyze somatic mutations, and so are more suitable for routine applications. Some of low-density platforms are commercially available [55–57], but none of them is specific focused to determine most clinically relevant mutations in melanoma patients.

Thus, there is a need to develop fast, inexpensive and highly sensitive method, based on low-density microarrays technology, which provides the detection of significant somatic mutations in melanomas to meet the demands for modern melanoma treatment. *BRAF, NRAS, KIT, MAP2K1/2, GNAQ* and *GNA11* mutations may be clinically useful for selecting patients for different targeted therapies. In the present study, we proposed a reliable biochip-based approach designed to simultaneously detect 39 recurrent mutations in melanoma genes. The high sensitivity has been reached by combining, in one assay, the LNA PCR clamp technique and the mutation-specific hybridization on a hydrogel biochip, which allows the identification of somatic mutations in a large excess of wild type (WT) DNA. Previously, this approach has been developed and applied for the analysis of somatic mutations in *EGFR, KRAS, BRAF, PI3K* genes in lung cancer cells [61].

This method was validated and used to analyze the frequency of mutations in 253 melanoma patients, using tumor-derived DNA from fresh-frozen and formalin-fixed paraffin-embedded (FFPE) tissues. The results obtained with this new approach were compared to the results obtained by traditional Sanger sequencing, NGS and ARMS/Scorpion real-time PCR.

## RESULTS

### Biochip assay for *BRAF, NRAS, KIT, MAP2K1/2, GNAQ* and *GNA11* mutation detection

**Figure 1:**
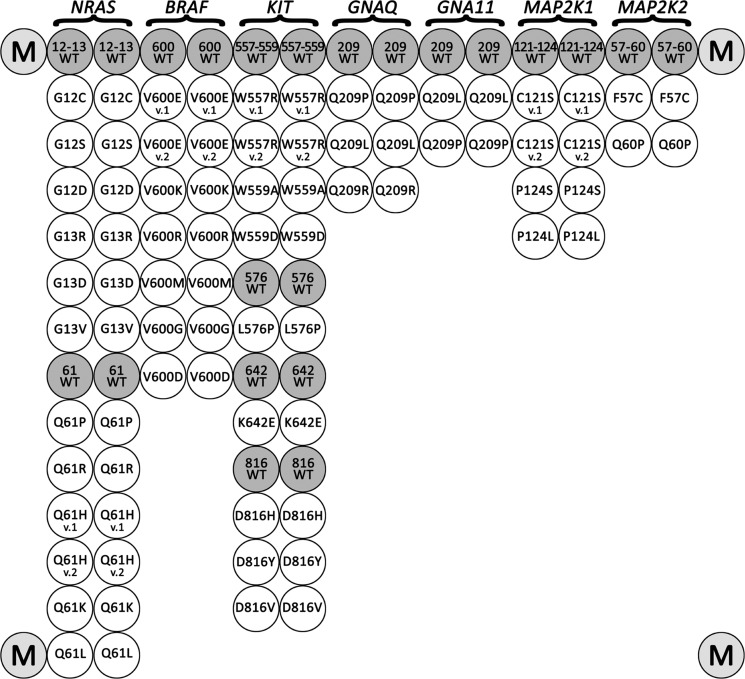
Biochip spotting scheme The biochip includes paired probes for the detection of each somatic mutations and corresponding wild-type sequences. Marker spots with Cy5 are located in the corners.

**Figure 2:**
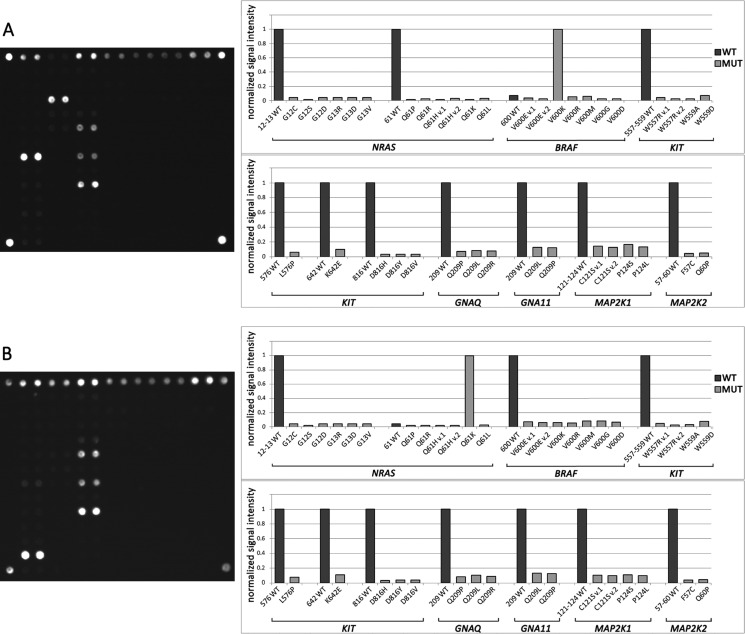
Mutational analysis by biochip (**A**) Biochip image and histogram of normalized signal intensity obtained for a sample with a V600K *BRAF* mutation. The bright spots in the hybridization image correspond to the outstanding bars on the histogram. All gel spots correspond to one analyzed site with common primers, and LNA oligonucleotides are combined into one group. Fluorescent signals from the paired probes are averaged. The fluorescent signal is normalized to the maximum signal in a group of gel spots. The sample contains a V600K *BRAF* mutation because *J*_(V600K)_*>J*_(600WT)_. (**B**) Biochip images and histograms of normalized signal intensity obtained for a sample with a Q61K *NRAS* mutation.

A scheme of the biochip is presented in Figure 1. The examples of hybridization patterns for samples with the V600K mutation of *BRAF* and a Q61K *NRAS* mutation are shown in Figure 2. The mutated sequences were predominantly amplified because the LNA PCR clamp was used. Insignificant wild-type sequence amplification occurred during the second round of PCR. Further, the amplified DNA fragments carrying the fluorescent label were bound to the oligonucleotide probes on the biochip; therefore, the fluorescent label was accumulated in the gel. The biochip assay was able to detect 39 different mutations in the *BRAF, NRAS, KIT, MAP2K1/2, GNAQ* and *GNA11* genes simultaneously.

Before using the biochip assay for clinical analysis, it was tested by an analysis of 40 control samples with known genotypes, which were characterized by direct sequencing as follows: WT sample and samples harbored all variations of the mutations localized on the biochip. All genotypes were identified by the biochips correctly.

### Sensitivity of the biochip assay

**Figure 3:**
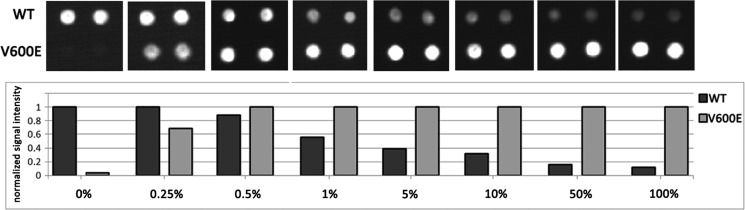
Biochip assay sensitivity The assay sensitivity for *BRAF* V600E mutation detection was determined by an analysis of serially diluted mutant DNA in the background of WT DNA: 0%, 0.25%, 0.5%, 1%, 5%, 10%, 50% and 100%. Fragment of a biochip images (in *the upper part*) and the normalized signal intensity (in *the lower part*) only for the spots corresponding to *BRAF* 600WT and V600E are present for each dilution.

The biochip assay sensitivity was determined by analyzing serially diluted mutant DNA (*BRAF* V600E) in a background of wild-type DNA in different ratios (0%, 0.25%, 0.5%, 1%, 5%, 10%, 50% and 100%). The samples were analyzed in triplicate. Our results showed that the biochip assay was able to detect 0.5% mutated DNA in wild-type DNA (Figure 3).

### Analysis of clinical samples by biochip assay

The clinical characteristics of the patients are reported in Table 1. In 177 of 253 (70.0%) melanoma patients, somatic mutations in the analyzed genes were detected by the biochip assay (Table 2): 129 (51.0%) *BRAF*, 45 (17.8%) *NRAS*, 6 (2.4%) *KIT*, 4 (1.6%) *GNAQ*, 2 (0.8%) *GNA11*, 2 (0.8%) *MAP2K1* and none of *MAP2K2*. The biochip allows the detection of only the most common somatic mutations, which are listed in Table 2.

**Table 1:** Patient clinical characteristics

Characteristic	No. of patients
**Age (median ± SD)**	53.7 ± 16.4
**Sex**	
Male	97
Female	140
NA (NA - not available)	16
**Location**	
Trunk	82
Upper limb	23
Lower limb	66
Head and Neck	30
NA	52
**Tumor subtype**	
superficial spreading melanoma	24
acral-lentiginuous	1
lentigo maligna	10
nodular	43
NA	175
**Stage**	
0	1
I A + B	14
II A + B	44
III A + B	17
IV	5
NA	172
**Breslow thickness**	
≤ 1 mm	26
1.01–2.0 mm	19
2.01–4.0 mm	28
> 4 mm	44
NA	136
**Clark level**	
I	3
II	13
III	65
IV	18
V	20
NA	133
**Ulceration**	
Yes	68
No	50
NA	135

**Table 2:** Analysis of the 253 melanoma patients using the biochip assay

Mutations	No. of patients (%)
***BRAF***	129 (51.0%)
V600E (c.1799T>A)	111
V600E (c.1799_1800delTGinsAA)	0
V600K (c.1798_1799delGTinsAA)	14
V600R (c.1798_1799delGTinsAG)	3
V600M (c.1798G>A)	1
V600G (c.1799T>G)	0
V600D (c.1799_1800delTGinsAT)	0
***NRAS***	45 (17.8%)
G12C (c.34G>T)	0
G12S (c.34G>A)	0
G12D (c.35G>A)	0
G13R (c.37G>C)	1
G13D (c.38G>A)	1
G13V (c.38G>T)	0
Q61P (c.182A>C)	0
Q61R (c.182A>G)	17
Q61H (c.183A>C)	2
Q61H (c.183A>T)	1
Q61K (c.181C>A)	20
Q61L (c.182A>T)	3
***KIT***	6 (2.4%)
W557R (c.1669T>A)	0
W557R (c.1669T>C)	0
V559A (c.1676T>C)	0
V559D (c.1676T>A)	0
L576P (c.1727T>C)	5
K642E (c.1924A>G)	1
D816H (c.2446G>C)	0
D816Y (c.2446G>T)	0
D816V (c.2447A>T)	0
***GNAQ***	4 (1.6%)
Q209P (c.626A>C)	2
Q209L (c.626A>T)	2
Q209R (c.626A>G)	0
***GNA11***	2 (0.8%)
Q209L (c.626A>T)	2
Q209P (c.626A>C)	0
***MAP2K1***	2 (0.8%)
C121S (c.361T>A)	0
C121S (c.362G>C)	0
P124S (c.370C>T)	2
P124L (c.371C>T)	0
***MAP2K2***	0 (0%)
F57C (c.170T>G)	0
Q60P (c.179A>C)	0
***WT***	76 (30.0%)

### Comparison with ARMS/Scorpion real-time PCR

The 98 melanoma samples were screened for *BRAF* V600E/K/R/D mutations by ARMS/Scorpion real-time PCR (BRAF RGQ PCR Kit, Qiagen, Germany). In 58 of 98 (59.2%) samples, somatic mutations in the *BRAF* gene were detected. Discordance with the biochip data was shown in 10 samples (Table 3). The specimens with discordant results were subjected to Sanger sequencing with and without the enrichment of mutant DNA by LNA PCR clamp. In 8/10 cases, the sequencing method (with or without the enrichment of mutant DNA) confirmed the biochip data. In 2 cases, the samples harbored the rare mutations V600V (c.1800G>A; COSM249890) and T599_V600insT (c.1797_1798insACA; COSM144982). The specific hybridization probes were absent on the biochip; therefore, the mutations could not be detected.

**Table 3:** Identification of *BRAF* mutations using the ARMS/Scorpion real-time PCR, biochip assay, sequencing and LNA PCR clamp with sequencing (samples that yielded discordant results are included only)

Patient No	Biochip	ARMS/Scorpion real-time PCR	Sanger sequencing	LNA PCR clamp + Sanger sequencing
163	V600E (c.1799T>A)	WT	V600E (c.1799T>A)	V600E (c.1799T>A)
191	V600K (c.1798_1799delGTinsAA)	WT	V600K (c.1798_1799delGTinsAA)	V600K (c.1798_1799delGTinsAA)
219	WT	V600R (c.1798_1799delGTinsAG)	WT	V600V (c.1800G>A)
223	WT	V600E (c.1799T>A)	WT	WT
226	WT	V600K (c.1798_1799delGTinsAA)	WT	WT
235	V600M (c.1798G>A)	WT	WT	V600M (c.1798G>A)
238	WT	V600E (c.1799T>A)	T599_V600insT (c.1797_1798insACA)	T599_V600insT (c.1797_1798insACA)
239	V600E (c.1799T>A)	V600K (c.1798_1799delGTinsAA)	WT	V600E (c.1799T>A)
241	WT	V600K (c.1798_1799delGTinsAA)	WT	WT
242	WT	V600K (c.1798_1799delGTinsAA)	WT	WT

### Comparison with Next generation sequencing

A total of 25 melanoma samples and 6 melanoma cell lines were tested by NGS (GS Junior, 454 Life Sciences, Branford, USA) (Table 4). In 4 samples, somatic mutations in the *BRAF* or *NRAS* genes were detected only by the biochip assay. In all cases, the results of the biochip assay were confirmed by sequencing with the enrichment of the mutant DNA by LNA PCR clamp. The NGS failed to detect these mutations, probably due to very low percentage of tumor cells in a sample, because the fresh-frozen tissue samples did not subjected to histological control before the analysis. In cell line Mel Cher a low percentage of cells carrying *NRAS* mutation may be explained by clonal heterogeneity or cross-contamination between different cell lines. In over 5 controversial cases, the genetic alterations were detected only by NGS. These cases represent germinal mutations and SNPs in the *KIT* and *BRAF* genes. The specific hybridization probes were absent on the biochip; therefore, the mutations could not be detected.

**Table 4:** Comparison NGS with biochip assay

Patient No	Biochip	NGS	Sanger sequencing	LNA PCR clamp + Sanger sequencing
2	*BRAF* V600E (c.1799T>A)	*BRAF* V600E (c.1799T>A)	*-*	-
31	WT	WT	-	-
52	*BRAF* V600E (c.1799T>A)	*BRAF* V600E (c.1799T>A)	-	-
91	WT	WT	-	-
92	*BRAF* V600E (c.1799T>A)	*BRAF* V600E (c.1799T>A)	-	-
93	*NRAS* Q61K (c.181C>A)	WT	WT	*NRAS* Q61K (c.181C>A)
94	*BRAF* V600E (c.1799T>A);*MAP2K1* P124S (c.370C>T)	*BRAF* V600E (c.1799T>A);*MAP2K1* P124S (c.370C>T)	-	-
95	*BRAF* V600E (c.1799T>A)	*BRAF* V600E (c.1799T>A)	-	-
96	*BRAF* V600E (c.1799T>A)	WT	-	-
97	*BRAF* V600E (c.1799T>A)	*BRAF* V600E (c.1799T>A)	-	-
98	WT	WT	-	-
99	*BRAF* V600K (c.1798_1799delGTinsAA)	*BRAF* V600K (c.1798_1799delGTinsAA)	-	-
100	*BRAF* V600E (c.1799T>A)	*BRAF* V600E (c.1799T>A)	-	-
101	WT	*KIT* M541L (c.1621A>C)	-	-
102	*BRAF* V600E (c.1799T>A)	*BRAF* V600E (c.1799T>A)	-	-
103	*NRAS* Q61H (c.182A>C)	*NRAS* Q61H (c.182A>C)	-	-
104	WT	WT	-	-
105	*BRAF* V600E (c.1799T>A)	*BRAF* V600E (c.1799T>A)	-	-
106	*BRAF* V600K (c.1798_1799delGTinsAA)	*KIT* V50L (c.148G>T)	WT	*BRAF* V600K (c.1798_1799delGTinsAA)
107	*BRAF* V600E (c.1799T>A)	*BRAF* V600E (c.1799T>A)	-	-
108	*NRAS* G13R (c.37G>C)	*NRAS* G13R (c.37G>C)	-	-
109	*NRAS* Q61K (c.181C>A)	*NRAS* Q61K (c.181C>A)	-	-
110	*BRAF* V600E (c.1799T>A)	*BRAF* V600E (c.1799T>A)	-	-
111	*BRAF* V600E (c.1799T>A)	*KIT* M541L (c.1621A>C);*BRAF* V600E (c.1799T>A)	-	-
112	*NRAS* Q61K (c.181C>A)	WT	WT	*NRAS* Q61K (c.181C>A)
cell line SK-MEL2	*NRAS* Q61R (c.182A>G)	*NRAS* Q61R (c.182A>G); *KIT*M541L (c.1621A>C), G245S (c.733G>A)	-	-
cell line Mel Il	*BRAF* V600K (c.1798_1799delGTinsAA)	*BRAF* V600K (c.1798_1799delGTinsAA);*BRAF* R389C (c.1165C>T)	-	-
cell line Mel Ibr	*BRAF* V600E (c.1799T>A)	*BRAF* V600E (c.1799T>A)	-	-
cell line Mel Rac	*NRAS* Q61R (c.182A>G)	*NRAS* Q61R (c.182A>G)	-	-
cell line Mel Cher	*NRAS* Q61R (c.182A>G)	WT	WT	*NRAS* Q61R (c.182A>G)
cell line Mel Z	*BRAF* V600E (c.1799T>A)	*BRAF* V600E (c.1799T>A)	-	-

Sequencing and LNA PCR clamp with sequencing were used us control methods

### Comparison with sanger sequencing

In total, 119 of the 253 samples were screened for *BRAF, NRAS, KIT, GNAQ, GNA11* and *MAP2K1/2* mutations by Sanger sequencing. In 87/119 samples, the genotype coincided with the data obtained by the biochip. In 2 (1.7%) cases, the rare mutations in the *BRAF* gene were identified by Sanger sequencing only: V600D (c.1799_1800TG>AC, COSM308550) and A598_T599insV (c.1794_1795insGTT, COSM26625). The 30 specimens with discordant results were subjected to LNA PCR clamp followed by sequencing. In 29/30 samples, the results were identical to the data obtained with the biochip assay. In 1 sample, a rare mutation, G60G (c.180A>T), was detected in the *NRAS* gene.

### Genetic alterations in melanoma patients

Summarizing the results obtained by all genotyping methods, 185/253 (73.1%) melanoma patients harbored somatic mutations in the analyzed genes.

### -*BRAF* mutations

**Figure 4:**
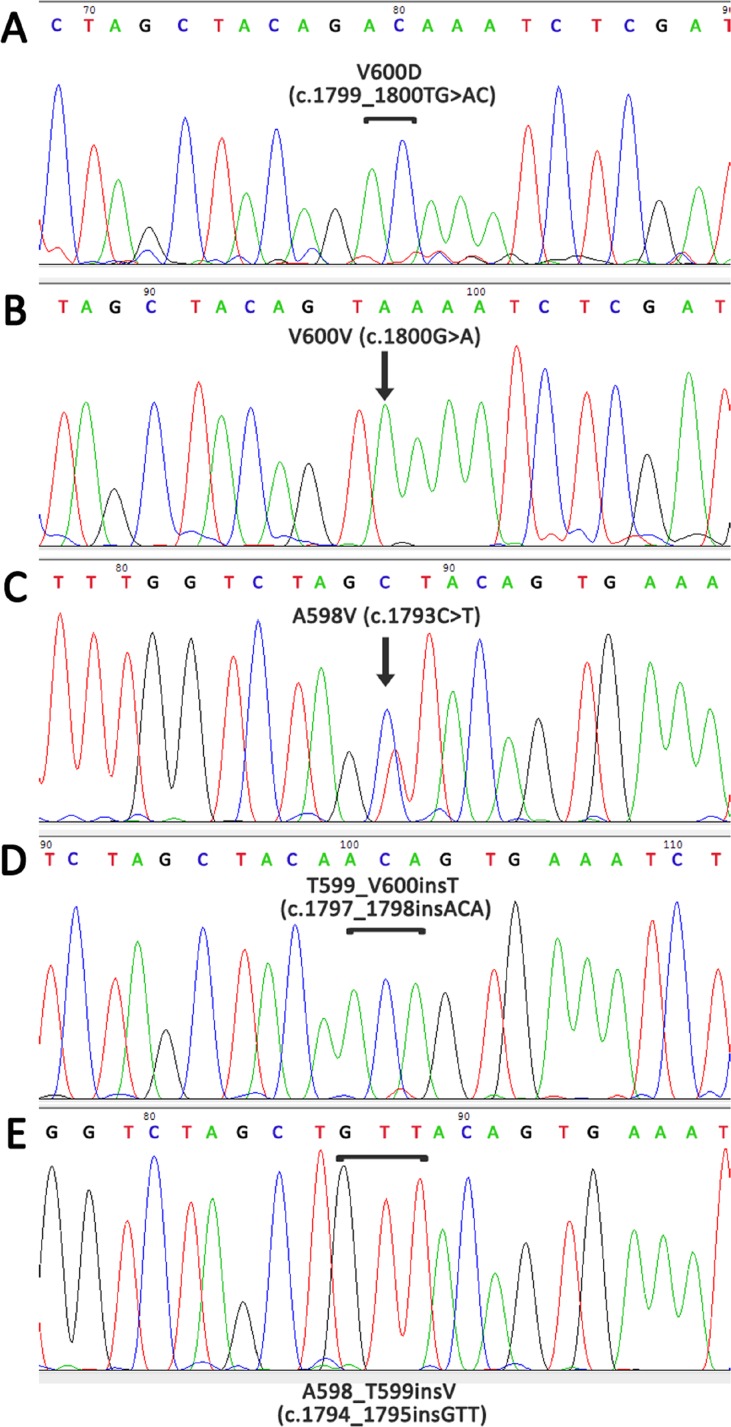
Detection of rare mutations in the *BRAF* gene using Sanger sequencing with preliminary enrichment with mutant allele (**A**) V600D, c.1799_1800TG>AC, COSM308550; (**B**) V600V, c.1800G>A, COSM249890; (**C**) A598V, c.1793C>T, COSM21549; (**D**) T599_V600insT, c.1797_1798insACA, COSM144982; (**E**) A598_T599insV, c.1794_1795insGTT, COSM26625.

**Figure 5:**
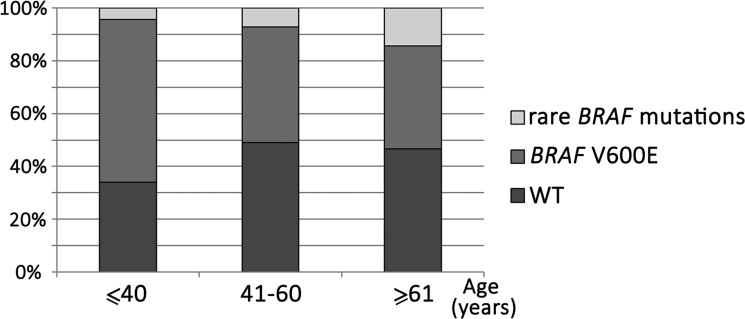
Frequency of rare *BRAF* mutations in different age groups of patients Rare mutations are more common in older patients (*P* = 0.05).

In total, 134/253 (53.0%) melanoma patients carried *BRAF* mutations. In most cases, the frequent mutations were identified using the biochip (111/134 [82.8%] V600E mutation, 14/134 [10.4%] V600K, 3/134 [2.2%] V600R, and 1/134 [0.7%] V600M). In five cases (5/253, 2.0%), we detected rare mutations only by Sanger sequencing (1/134 [0.7%] V600D (c.1799_1800TG>AC, COSM308550), 1/134 [0.7%] V600V (c.1800G>A, COSM249890), 1/134 [0.7%] A598V (c.1793C>T, COSM21549), 1/134 [0.7%] A598_T599insV (c.1794_1795insGTT, COSM26625), 1/134 [0.7%] p.T599_V600insT (c.1797_1798insACA, COSM144982) (Figure 4). Thus, 17.2% of *BRAF*-mutated patients showed a rare mutation (non-V600E). The frequency of rare *BRAF* mutations increased with age (*P* = 0.05). Only 4.3% of patients ≤ 41 years old harbored non-V600E mutation, while 14.4% of patients ≥ 61 years old were non-V600E (Figure 5).

Women showed a higher frequency of *BRAF* mutations compared to men (85/140 [60.7%] vs. 46/97 [47.4%], *P* = 0.047). The *BRAF* mutated patients, compared with the WT *BRAF* patients, showed a slightly lower median age as follows: 52.7±14.2 years (range 17-83 years) vs. 55.1±18.8 years (range 0.1–88 years) (*P* = 0.108). There were no significant associations among the *BRAF* mutation status, tumor localization, subtype, stage, Breslow thickness, Clark level or ulceration.

### -*NRAS* mutations

*NRAS* mutations were found in 45/253 (17.8%) patients. In one case, the patient harbored two mutations, Q61K and G60G (c.180A>T), in the *NRAS* gene simultaneously. In total, 43/46 (93.4%) of these mutations were observed in codon 61 as follows: 20/46 (43.5%) Q61K, 17/46 (37.0%) Q61R, 3/46 (6.5%) Q61L, 2/46 (4.3%) Q61H (c.183A>C) and 1/46 (2.2%) Q61H (c.183A>T) mutations. Mutations in codon 13 were detected only in 2 patients as follows: 1/46 (2.2%) G13R and 1/46 (2.2%) G13D. The rare mutation G60G (c.180A>T) was detected only by Sanger sequencing.

The *NRAS* mutations occurred more frequently in men than in women; however, the difference was not statistically significant (22/97 [22.7%] vs. 19/140 [13.6%], *P* = 0.0813). *NRAS* mutations were more common in nodular melanoma than in superficial spreading melanoma (7/43 [16.3%] vs. 0/24 [0%], *P*=0.0367). There were no associations between tumor *NRAS* mutation status, location, age, stage, Breslow thickness, Clark level or ulceration.

### -*KIT* mutations

Six (2.4%) patients had *KIT* mutations. Five of these patients harbored the mutation in codon 576 (83.3%), and one (16.7%) harbored the mutation in codon 624 (K642E). The *KIT* mutation status did not correlate with gender, age or ulceration.

### -*GNAQ* mutations

The *GNAQ* mutations in codon 209 were detected in 2.0% (5/253) of our melanoma cases. The Q209P (c.626A>C) and Q209L (c.626A>T) mutations were identified in 2 cases, and the Q209L (c.625_626CA>TT) mutation was identified in 1 case. Q209L (c.625_626CA>TT) is a rare mutation detected only by Sanger sequencing. Mutations in the *GNAQ* gene were not associated with age, gender or ulceration.

### -*GNA11* mutations

The Q209L *GNA11* gene mutation was detected in 2 (0.8%) of 253 patients. In both cases, the mutations were identified in females. The *GNA11* mutations were mutually exclusive, with mutations in the *BRAF*, *NRAS*, *KIT*, *GNAQ* and *MAP2K1/2* genes. Mutations in the *GNA11* gene were not associated with age or gender. The other correlations were invalid due to the small number of analyzed groups.

### -*MAP2K1* mutations

The P124S *MAP2K1* gene mutation was found in 2/253 (0.8%) patients. In both cases, the *MAP2K1* mutation was identified in tumors simultaneously harboring the *BRAF* V600E mutation. *MAP2K1* mutation status did not correlate with age, gender or ulceration.

### -*MAP2K2* mutations

The F57C and Q60P mutations in the *MAP2K2* gene were not found in the 253 melanoma patients.

## DISCUSSION

The mutation status of a tumor is critical for considering targeted therapy for patients with melanoma. In this study, we proposed a biochip-based assay for the detection of somatic mutations in melanoma. Although several methodologies are currently used to test somatic mutations (Sanger sequencing, NGS, PCR-related technologies and others), many of them have flaws in comparison to the biochip assay. Sanger sequencing has a relatively low sensitivity with a detection limit of approximately 15–30% mutant alleles [62, 63]. NGS is becoming more widely adopted as a valuable method for somatic mutation analysis in cancer. NGS offers high sensitivity and accurate data quality for identifying even rare mutations successfully. These advantages are driving increased adoption of NGS in clinical cancer research, but this method is time-consuming and, thus far, rather expensive.

The real-time PCR based commercial kits, such as the COBAS 4800 BRAF V600 Mutation Test (Roche Molecular Diagnostics, Pleasanton, USA) and BRAF RGQ PCR Kit (TheraScreen, Qiagen, Hilden, Germany), are used worldwide and have sensitivity levels of approximately 5% but are expensive, allowing for the identification of a limited number of mutations.

Microarrays remain useful and accurate tools for parallel analysis of actionable mutations in cancer. The Affymetrix OncoScan array has been optimized for whole genome copy number, loss of heterozygosity and somatic mutation detection from highly degraded FFPE samples. Based on molecular inversion probe technology, the assay currently detects 74 somatic mutations commonly found in 9 cancer genes (*BRAF*, *KRAS*, *EGFR*, *IDH1*, *IDH2*, *PTEN*, *PIK3CA*, *NRAS* and *TP53*). This high-density platform is reliable and powerful, but is rather labor-consuming and requires expensive equipment [53, 54]. The claimed sensitivity of the assay in relation to variant allele frequency is about 5%, which may be not enough while working with the bulk of the tumor tissue [64]. Although the low-density microarrays allow to analyze much less genes, they are more suitable for routine use due to simple laboratory procedure, reliability and low cost of analysis [55–60].

We proposed the biochip-based assay, which combined multiplex LNA PCR clamp followed by hybridization with an array of allele-specific immobilized probes, allowing for the identification of 39 mutations in the *BRAF, NRAS, KIT, MAP2K1/2, GNAQ* and *GNA11* genes. Our data showed that this approach could detect at least 0.5% of mutated sequences in a background of WT DNA. The biochips are inexpensive (approximately $15 per chip) and may be useful as a routine laboratory test. The workflow included isolation of DNA from fresh frozen or FFPE tumor, LNA PCR clamp, hybridization on the biochip and image analysis and requires no more than 20–24 h. Twenty-four clinical samples could be tested in parallel. To reach the highest accuracy of the mutation analysis, verification of the hybridization results may be recommended using the same primer set in LNA PCR clamp, followed by Sanger sequencing.

In our study, 253 melanoma patients were analyzed by developing a biochip-based approach. Mutations in the *BRAF, NRAS, KIT, GNAQ, GNA11* and *MAP2K1* genes were found in 51.0%, 17.8%, 2.4%, 1.6%, 0.8% and 0.8% of the cases, respectively. Mutations in the *MAP2K2* gene were not detected.

The biochip-based assay was compared with the following three widely used methods: NGS, ARMS/Scorpion real-time PCR and Sanger sequencing.

In this study, we analyzed 25 melanoma patients and 6 melanoma cell by NGS and biochip assay in parallel. Despite a 100-fold coverage in NGS, in 4 samples somatic mutations in *BRAF* or *NRAS* genes were detected only by the biochip assay. The results were confirmed by Sanger sequencing with the preliminary enrichment of mutant DNA. In 5 cases, the genetic alterations were detected only by NGS and represented allelic variants in the *KIT* and *BRAF* genes, which were not considered valuable for choice of therapy. Thereby, the biochip assay was not inferior to the NGS approach in the detection of clinically relevant driver mutations in melanoma samples.

The 98 melanoma samples were screened for *BRAF* mutations by ARMS/Scorpion real-time PCR and hybridization with biochips. The mutation rate of ARMS/Scorpion real-time PCR in these samples was 59.2% compared with 56,1% detected by biochips. Discordance in the results was obtained in 10 samples. In 1/10 case, the two methods showed different types of mutations. In 3/10 cases, we detected mutations only by biochip assay. In 6/10 cases, we detected mutations only by the BRAF RGQ PCR Kit. The specimens with discordant results were subjected to Sanger sequencing (with and without the enrichment of mutant DNA), and, in 8/10 cases, sequencing confirmed the biochip data. In 2/10 cases, samples harbored rare *BRAF* mutations, V600V and T599_V600insT, which were not included in the biochip and, therefore, could not be detected.

The biochip assay was compared with Sanger sequencing. The biochip allowed detecting somatic mutations in 70% of samples, while the Sanger sequencing without a preliminary enrichment by mutant DNA revealed mutations only in 53% of cases. The sequencing approach with preliminary enrichment by mutant allele using LNA-clamp PCR made possible the identification of mutations in a high percentage of cases similar to biochip assay.

Based on these results, we can conclude that the biochip-based assay is a reliable, accurate, reproducible and highly sensitive method for the detection of frequent mutations in melanoma.

In total, somatic mutations in the *BRAF, NRAS, KIT, GNAQ, GNA11*, *MAP2K1* and *MAP2K2* genes were found in 53.0%, 17.8%, 2.4%, 2.0%, 0.8%, 0.8% and 0% of the 253 cases, respectively. These frequencies are very close to those previously reported in melanoma patients [23, 35, 65–67]. In general, *BRAF*, *NRAS, GNAQ* and *GNA11* mutations were not found in one tumor sample. Moreover, we found that mutations in *BRAF* with *NRAS* (*P* < 0.0001) and *BRAF* with *GNAQ* (*P* = 0.022) are mutually exclusive. In contrast, *MAP2K1* mutations were present together with mutations in the *BRAF* gene.

In addition, in this study, we defined a relationship between the mutation status and the clinical characteristics of the patients. In our study, the age of *BRAF* mutated patients was found to be slightly lower than *BRAF* WT patients (*P* = 0.108). However, in many studies, this difference was statistically significant [68–70]. The lack of significant difference in the two groups of patients might be explained by the inclusion of children with melanoma in our study. By analyzing only the adult patients (over 21 years), we obtained a statistically significant difference between the MUT and WT *BRAF* groups (53.21 ± 13.59 vs. 58.51 ± 13.99, *P* = 0.0148).

The higher proportion of non-V600E genotypes in *BRAF*-mutated melanomas was observed in older patients (*P* = 0.05). This trend is in accordance with the publication of Menzies et al. [71].

Women showed a higher frequency of *BRAF* mutations compared to men (85/140 [60.7%] vs. 46/97 [47.4%], *P* = 0.047), but other studies did not demonstrate statistically significant associations between *BRAF* mutation status and gender [70, 72, 73].

The *NRAS* mutations occurred more frequently in men than in women; however, the difference was not statistically significant (22/97 [22.7%] vs. 19/140 [13.6%], *P* = 0.0813) in our study and other studies [74, 75]. *NRAS* mutations were more common in nodular melanoma than in superficial spreading melanoma (7/43 [16.3%] vs. 0/24 [0%], *P* = 0.0367). Similar data were obtained in other studies [76–78], but several groups found no difference in *NRAS* mutation rates among nodular and superficial spreading melanoma [79, 80].

In general, the association between the tumor genotype of patients and their clinical characteristics also corresponds to previously published data.

In this study, we proposed a highly sensitive, reproducible and fast method for *BRAF, NRAS, KIT, GNAQ, GNA11* and *MAP2K1/2* mutation detection based on a hydrogel biochip platform. This approach is easy-to-use, inexpensive and does not require expensive equipment. The mutation detection rate using this approach is as great as 70% in melanoma patients. The method is suitable for rapid and effective mutation screening in large research centers and small distant laboratories.

We have compared this method with NGS, ARMS/Scorpion real-time PCR and Sanger sequencing and concluded that the biochip-based approach has a great potential for routine clinical application and can be used in a rapid screening for the presence of somatic mutations in melanoma patients prior to targeted therapy appointment.

## MATERIALS AND METHODS

### Tumor samples and cell lines

The tumor samples were obtained from 253 melanoma patients who were treated at the Blokhin Cancer Research Center, Ministry of Health of the Russian Federation between 1984 and 2016 and the P. Hertsen Moscow Oncology Research Institute between 2003 and 2016. The mean age of the melanoma patients was 53.7 ± 16.4 years. All patients provided written informed consent. Ethical approval for the study was provided by the Ethical Committees of the Blokhin Cancer Research Center and the P. Hertsen Moscow Oncology Research Institute.

The tumor tissues were collected by surgical resection. In total, 63/253 tissue samples were frozen directly after surgery and were stored at −20°C until DNA extraction, 190/253 tissue samples were fixed in formalin and embedded in paraffin.

In addition, genomic DNA was derived from the following 6 melanoma cell lines: Mel Rac [81], Mel Z [82], Mel Cher [83], Mel Il [84], Mel Ibr [85] and SK-MEL-2 (ATCC^®^ HTB-68^™^, Guernsey, Ireland, United Kingdom).

### Biochip assay

The procedure of the biochip approach analysis consisted of the following successive steps: (1) DNA isolation from the tumor tissue; (2) LNA clamped multiplex PCR to predominant amplification of the mutant DNA in the presence of large excess of wild-type DNA; (3) asymmetric multiplex nested PCR to yield fluorescently labeled single-stranded DNA fragments; (4) hybridization of labeled PCR-products on a biochip; and (5) hybridization image analysis.

### DNA isolation

Genomic DNA from the tumor tissues was isolated using a QIAamp DNA Micro Kit, QIAamp DNA FFPE Tissue Kit (Qiagen, Hilden, Germany) or blackPREP FFPE DNA Kit (Analytik Jena, Jena, Germany) according to the manufacturer's instructions. The quantity of nucleic acids was controlled by the Qubit fluorometer (Invitrogen, Life Technologies Corporation, Carlsbad, USA).

### Oligonucleotide probes and biochip fabrication

Oligonucleotide probes for immobilization on the biochip (Supplementary Table 1) were synthesized on a 394 DNA/RNA synthesizer (Applied Biosystems, Carlsbad, USA) using standard phosphoramidite chemistry. All oligonucleotides carry an amino group at the 5′-terminus for immobilization in the polyacrylamide gel. The amino group was introduced during synthesis using a 3′-amino-modifier C7 CPG 500 (Glen Research, Sterling, USA). Some oligonucleotides included the LNA residues (Exiqon, Vedbaek, Denmark) in addition to the DNA residues to increase the sensitivity and specificity of the hybridization. Microarrays of polyacrylamide gel drops were prepared using a copolymerization method [86]. Each gel drop is duplicated to improve the reliability of the analysis.

Primers for amplification (Supplementary Table 2) were synthesized commercially (Evrogen, Moscow, Russian Federation).

We used LNA-containing clamping oligomers, which consisted of LNA and DNA residues or LNA residues only (Supplementary Table 3). The 3′-terminus was phosphorylated to prevent extension by Taq-polymerase. LNA-oligomers were synthesized on a 394 DNA/RNA synthesizer.

### Preparation of DNA targets for hybridization

Target DNA samples were prepared through four parallel nested (two-round) multiplex PCR reactions. The first reaction was used to amplify *KIT* (codons 576 and 816) fragments; the second reaction was used to amplify *BRAF* and *NRAS* fragments; the third reaction was used to amplify fragments of *KIT* (codons 557 and 559, 642); and the fourth reaction was used to amplify the *GNAQ*, *GNA11*, *MAP2K1* and *MAP2K2* loci. The SNPdetect polymerase was used for the amplification because it does not have 5′ to 3′ exonuclease activity (Evrogene, Moscow, Russian Federation). The multiplex PCR was performed in a total volume of 12 μl, containing 1× SNPdetect buffer, 2.5 units SNPdetect polymerase, 0.2 mM dNTPs, 0.2 μM primers, 0.02–0.2 μM LNA-oligomers (the optimal concentration for each LNA-oligonucleotide was selected individually) and 5–12 ng genomic DNA. The cycling conditions were as follows: for 3 min and 30 s at 94^°^C, followed by 35 cycles of 30 s at 94^°^C, 20 s at 60^°^C, 10 s at 72^°^C and a final elongation at 72^°^C for 3 min. The PCR was performed on a T100 thermal cycler (Bio-Rad, Hercules, USA).

Each multiplex PCR (12 μl) in the second round contained 1× SNPdetect buffer, 2.5 units SNPdetect polymerase, 0.2 mM dNTPs, 0.2 μM forward primers and 2 μM reverse primers, 0.2 nM Cy5-dTTP and 1 μl of the first-round PCR product. The same program was used for the amplification. Thus, single-stranded fluorescently labeled PCR products were obtained.

### Hybridization and image analysis

The hybridization and image analysis were performed as described before [61]. Briefly, hybridization was performed in 40 μl of 25% formamide (Serva, Oklahoma City, USA), 6× saline-sodium phosphate-EDTA (Promega, Fitchburg, USA) and 20 μl lebeled PCR products. The hybridization mixture was denatured at 95°C (5 min), briefly cooled on ice (2 min) and then applied on a biochip under a hybridization chamber and left overnight at 37°C. The chamber was disassembled, and the biochip was washed for 10 min with 50 ml of 1× saline-sodium phosphate-EDTA at room temperature and then dried. Hybridization signals were monitored by a portable chip analyzer (BIOCHIP-IMB, Moscow, Russian Federation). Data processing and image analysis were performed using dedicated software ImaGeWare version 3.5 (BIOCHIP-IMB, Moscow, Russian Federation). The fluorescence signals produced by the biochip's gel spots were used as the input data as follows: *J*_m_ *= (I*_m_ *− I*_0_*)/(B*_m_ *− I*_0_), where *I*m is the signal intensity per unit area in the internal region of a gel pad, *B*m is the counterpart background intensity, *I*_0_ is the dark current in the charge-coupled device and *m* is the gel pad number. Because the gel spots were duplicated, the signal intensity was averaged. The assignment of mutation in a sample can be done if *J*_(mut)_ >*J*_(wt)_ and *J*_(mut)_ / *B*_m_ ≥ 2.

### Methods for validation

#### Sanger Sequencing

Sanger sequencing was used as a routine reference method for detecting mutations in the *BRAF*, *NRAS*, *KIT*, *GNAQ*, *GNA11*, *MAP2K1* and *MAP2K2* genes. For the identification of mutations in samples with low percentages of mutant DNA, we used LNA PCR clamp amplification followed by direct Sanger sequencing. The primers and LNA-oligomers for the sequencing analysis are shown in Supplementary Tables 4 and 3, respectively. For the amplification of most loci, we used the same primers that were used in the first-round of PCR for the biochip analysis.

PCR products were purified by ethanol precipitation of DNA with ammonium acetate and sequenced with the BigDye^TM^ Terminator 3.1 Cycle Sequencing Kit (Life Technologies Corporation, Carlsbad, USA) on an Applied Biosystems 3730 DNA Analyzer (Life Technologies Corporation, Carlsbad, USA) according to the manufacturer's instructions. The sequencing results were interpreted using Chromas Lite software V.2.1 (Technelysium Pty, Helensvale, Australia).

#### Next generation sequencing

NGS (GS Junior, 454 Life Sciences, Branford, USA) was used to analyze mutations in 25 melanoma samples and 6 melanoma cell lines. NimbleGen technology (Roche, Basel, Switzerland) was used to target regions enrichment, including *BRAF, NRAS, KIT, MAP2K1* and *MAP2K2* genes.

A bioinformatic analysis of the results was performed using the GS Reference Mapper. Sequence reads were aligned to the human reference genome (HG19). Sequence coverage was, on average, 100-fold for target regions. Variants obtaining a frequency of detection ≥ 10% were considered for the analysis.

#### ARMS/Scorpion real-time PCR

A BRAF RGQ PCR Kit (Qiagen, Hilden, Germany) was used according to the manufacturer's instructions. This assay is designed to detect a wild-type control and the four most common *BRAF* mutations V600E/K/R/D. Real-time PCR was performed on a Rotor-Gene Q Real-time PCR Platform (Qiagen, Hilden, Germany). The cycling conditions for quality control runs and mutation assays were as follows: 15 min at 95°C, followed by 40 cycles at 95°C for 30 s and 60°C for 1 min. Fluorescence was measured at 60°C. Data on each mutation were interpreted according to the kit manual after a curve analysis and calculation of ΔCt values.

### Statistical analyses

The χ^2^ with Yates correction and two-sided Fisher exact tests were used to determine correlations between the qualitative clinicopathologic characteristics and mutation status of the patients. The Mann-Whitney *U* test was used to analyze the relationship between age and mutation status. All statistical tests were two-sided. A *P*-value of 0.05 was the statistical significance threshold. The GraphPad InStat, version 3.05 (GraphPad Software Inc., San Diego, USA) was used for all analyses.

## SUPPLEMENTARY TABLES





## Abbreviations

LNA: locked nucleic acid
NGS: next generation sequencing
WT: wild type
FFPE: formalin-fixed paraffin-embedded
PCR: polymerase chain reaction
