# Supplementary material for: Detection of BRAF, NRAS, KIT, GNAQ, GNA11 and MAP2K1/2 mutations in Russian melanoma patients using LNA PCR clamp and biochip analysis
Source: Oncotarget. 2017 Apr 10;8(32):52304–20. doi: 10.18632/oncotarget.17014 (PMC5581030; doi:10.18632/oncotarget.17014)
Supplement: Supplementary file 1 [file oncotarget-08-52304-s001.pdf]

Detection of *BRAF*, *NRAS*, *KIT*, *GNAQ*, *GNA11* and *MAP2K1/2* mutations in Russian melanoma patients using LNA PCR clamp and biochip analysis

Supplementary Materials

Supplementary Table 1: Oligonucleotides for immobilization on the biochip. See_Supplementary_Table 1.

Supplementary Table 2: Primers for the multiplex amplification

Name[a]	Sequence (from 5′ to 3′)
BRAF 600-F1,F2	CTCTTCATAATGCTTGCTCTGATAG
BRAF 600-R1	TGATGGGACCCACTCCATC
BRAF 600-R2	GGACCCACTCCATCGAGAT
NRAS 12-13-F1,F2	CTTGCTGGTGTGAAATGACTGA
NRAS 12-13-R1	TGGGATCATATTCATCTACAAAGTG
NRAS 12-13-R2	GATTAGCTGGATTGTCAGTGC
NRAS 61-F1,F2	TGAAACCTGTTTGTTGGACATACT
NRAS 61-R1	GGCAAATACACAGAGGAAGCC
NRAS 61-R2	CCTTCGCCTGTCCTCATGTA
KIT 557-559-F1,F2	CAGAGTGCTCTAATGACTGAGA
KIT 557-559-R1	ACATAATTGTTTCCATTTATCTCCT
KIT 557-559-R2	GTTGGGTCTATGTAAACATAATTGTTTC
KIT 576-F1	GGTTGTTGAGGAGATAAATGGA
KIT 576-F2	GGAAACAATTATGTTTACATAGACC
KIT 576-R1,R2	GCCCCTGTTTCATACTGAC
KIT 642-F1	TCCAATTTTAGCGAGTGCC
KIT 642-F2	AGTGCCCATTTGACAGAAC
KIT 642-R1	CTTGGACACGGCTTTACCT
KIT 642-R2	GCAGGCTCCAAGTAGATTCA
KIT 816-F1,F2	CCTTACTCATGGTCGGATCA
KIT 816-R1	AATCCTTTGCAGGACTGTC
KIT 816-R2	GAGAATGGGTACTCACGTTTC
GNAQ 219-F1,F2	GAGTATTGTTAACCTTGCAGAATGGT
GNAQ 219-R1	AGCGCTACTAGAAACATGATAGAGGT
GNAQ 219-R2	CGCTACTAGAAACATGATAGAGGTGAC
GNA11 219-F1,F2	GCTGTGTCCTTTCAGGATGGT
GNA11 219-R1	GAGAAACATGATGGATGTCACGTT
GNA11 219-R2	TGTCACGTTCTCAAAGCAGTG
MAP2K1 121-124-F1,F2	GATCATAAGGGAGCTGCAGGTT
MAP2K1 121-124-R1	GAACGCACCATAGAAGCCCA
MAP2K1 121-124-R2	CGCACCATAGAAGCCCACG
MAP2K2 57-60-F1,F2	GAACTTGACGAGCAGCAGAAGA
MAP2K2 57-60-R1	CGAAGTCATCGTCTTTGAGTTCG
MAP2K2 57-60-R2	GTCATCGTCTTTGAGTTCGCC

[a]F1-forward primer of the first round of PCR; R1-reverse primer of the first round; F2-forward primer of the second round; R2-reverse primer of the second round; F1,F2- forward primer of the first and second rounds; R1,R2- forward primer of the first and second rounds.

Supplementary Table 3: LNA-oligomers were used for LC-biochip analysis

Name	Sequence (from 5′ to 3′)[a]
BRAF 600-LNA	5′-agatttcactgtag-PH-3′
NRAS 12-13-LNA	5′-agcaggtggtgttg-PH-3′
NRAS 61-LNA	5′-ctcttcttgtccagc-PH-3′
KIT 557-559-LNA	5′-aacaaccttccactg-PH-3′
KIT 576-LNA	5′-AtaAggaagttgTgt-PH-3′
KIT 642-LNA	5′-gaACtcaaagtcct-PH-3′
KIT 816-LNA	5′-ttgatgtctctggcT-PH-3′
GNAQ 209-LNA	5′-GGGgCcaaaGGtCag-PH-3′
GNA11 209-LNA	5′-GGgGCcagCGGtCG-PH-3′
MEK1 121-124-LNA	5′-GagtgCaaCTCTccgta-PH-3′
MEK2 57-60-LNA	5′-ctttctCacCcagAA-PH-3′

[a]DNA-nucleotide is identified in upper case letters, and LNA-nucleotide is identified in lower case letters, PH-phosphate group.

Supplementary Table 4: Primers for the sequencing

Name	Sequence (from 5′ to 3′)
BRAF_600_F seq	CTCTTCATAATGCTTGCTCTGATAG
BRAF_600_R seq	TGATGGGACCCACTCCATC
NRAS_12-13_F seq	CTTGCTGGTGTGAAATGACTGA
NRAS_12-13_R seq	CAGAATATGGGTAAAGATGATCCGA
NRAS_61_F seq	AACCTTGGCAATAGCATTGCATT
NRAS_61_R seq	GGCAAATACACAGAGGAAGCC
KIT_557-559_F seq	CAGAGTGCTCTAATGACTGAGA
KIT_557-559_R seq	GCCCCTGTTTCATACTGAC
KIT_576_F seq	CAGAGTGCTCTAATGACTGAGA
KIT_576_R seq	GCCCCTGTTTCATACTGAC
KIT_642_F seq	TCCAATTTTAGCGAGTGCC
KIT_642_R seq	CTTGGACACGGCTTTACCT
KIT_816_F seq	CCTTACTCATGGTCGGATCA
KIT_816_R seq	AATCCTTTGCAGGACTGTC
GNAQ_209_F seq	CATCGTCATTCAAGAGAATATTTTCC
GNA11_219_R seq	GAGAAACATGATGGATGTCACGTT
GNA11_209_F seq	AGCCGATGTCAGTCTGGTGTG
GNA11_219_R seq	GAGAAACATGATGGATGTCACGTT
MAP2K1_121-124_F seq	TGACACTCATTTCTTGGTTGAGCA
MAP2K1_121-124_R seq	ATGCAGATACTGATCTCGCCATC
MAP2K2_57-60_F seq	CACATAGCAAACGGCCAGA
MAP2K2_57-60_R seq	CGAAGTCATCGTCTTTGAGTTCG
